# Cassava processing residues as circular energy feed ingredients for dairy cows: variability, safety constraints, and technology-enabled stabilization strategies

**DOI:** 10.14202/vetworld.2026.3205-3230

**Published:** 2026-07-21

**Authors:** Budi Wardiman, Syahriani Syahrir, Asmuddin Natsir, Tilawati Tilawati, Aurelya Yulyanti Sudarmanto, J. Nurwahidah

**Affiliations:** 1 Department of Agricultural Science, Hasanuddin University, Makassar 90245, Indonesia.; 2 Department of Animal Husbandry, Faculty of Science and Technology, Bulukumba Muhammadiyah University, Bulukumba 92561, Indonesia.; 3 Feed Chemistry Laboratory, Faculty of Animal Husbandry, Hasanuddin University, Makassar 90245, Indonesia.

**Keywords:** aerobic stability, cassava processing residues, circular bioeconomy, cyanide detoxification, dairy cows, ensiling, feed safety, tropical dairy systems

## INTRODUCTION

Dairy production systems are increasingly expected to deliver high-quality animal-source foods while reducing their dependence on human-edible feed resources and lowering environmental burdens throughout the supply chain [1]. Within the broader circular bioeconomy framework, livestock, particularly ruminants, are often positioned as biological upcyclers that convert heterogeneous, low-opportunity-cost biomass into nutrient-dense foods while returning nutrients to agroecosystems through manure management [2]. In parallel, growing interest in agro-industrial co-products and food system residues has shifted the discussion from general sustainability narratives toward practical questions: which residue streams are nutritionally suitable, how variable are they, what hazards accompany them, and what processing and quality control measures are required for safe and consistent use in dairy production.

Among regionally abundant residue streams in tropical and subtropical agri-food systems, cassava (*Manihot esculenta*) processing residues have attracted renewed interest as potential energy sources for ruminant diets. Cassava production remains concentrated in tropical regions, and the scale of residue generation is substantial. In 2024, Africa and Asia produced approximately 222.84 and 90.8 million tons of cassava, respectively, while review-based estimates indicate that solid cassava wastes may reach about 50 million tons annually in Africa alone. These figures highlight cassava residues as a regionally abundant biomass resource with potential value for livestock feeding [3]. Recent syntheses highlight cassava’s relevance to ruminant feeding in many low- and middle-income regions due to its agronomic adaptability and high carbohydrate content, alongside growing emphasis on value-chain approaches that enable broader utilization in livestock systems [4]. Importantly, cassava waste is not a single material; it encompasses distinct streams generated along processing pathways (e.g., cassava pulp/pomace from starch extraction, peels, and other residues), each with different nutritional profiles and risk characteristics [5–7]. The dairy sector has a particular incentive to evaluate these streams because concentrate costs strongly influence the economics of milk production and because localized residue availability may buffer feed supply volatility [8]. This review focuses primarily on tropical and subtropical dairy systems, especially smallholder and cooperative settings where cassava residues are regionally abundant and wet handling is common, while also noting implications for more commercial, high-producing herds when residue streams are adequately standardized and stabilized [9, 10]. However, the practical integration of cassava processing residues into dairy rations remains constrained because variability, safety hazards, and rapid spoilage are often discussed separately rather than integrated into an operational framework for procurement, stabilization, and risk management.

A first constraint is compositional and physical variability, which is amplified when residues are produced as high-moisture materials that deteriorate rapidly under warm climates. In practice, cassava pulp may range from relatively starch-rich to more fibrous fractions depending on processing efficiency, dilution, and contamination with soil or fibrous materials; such variability complicates diet formulation and increases the risk of inconsistent rumen fermentation outcomes if not managed through specification-based procurement and blending strategies [11–13]. The circular feed literature emphasizes that variability is intrinsic to many residue streams, and that successful valorization depends on aligning processing technologies, quality assurance, and end use specifications rather than treating residues as nutritionally equivalent substitutes for conventional cereals [14]. For dairy systems, where rumen health and milk components are sensitive to carbohydrate fermentability and fiber effectiveness, variability management is not a secondary issue but a central determinant of feasibility.

A second, and distinctive, constraint is safety associated with cyanogenic glycosides in cassava-derived materials. Cyanogenic glycosides can release hydrogen cyanide (HCN) under specific conditions, posing toxicity risks that limit cassava utilization without appropriate processing and monitoring [15]. Recent work has increasingly shifted from simply describing the presence of cyanide toward evaluating practical mitigation methods and their effectiveness under farm-relevant conditions. For example, ensiling of cassava materials has been investigated not only as a preservation strategy but also as a detoxification approach, with evidence that fermentation conditions and microbial interventions can influence residual cyanide levels and associated rumen fermentation responses [16–19]. This creates a critical opportunity for dairy nutrition: cassava residues may be rendered safer and more stable through targeted processing, but only if the control points (e.g., chopping, moisture management, fermentation dynamics, aerobic stability) are systematically integrated into handling and feeding protocols.

A third constraint involves nutritional “fit” within high-producing dairy diets, where the margin for error is narrow. Cassava residues are typically considered energy contributors, yet their fermentability characteristics and low intrinsic protein concentration (particularly for pulp streams) mean that substitution decisions have to account for synchrony between rapidly fermentable carbohydrates and rumen-degradable nitrogen, as well as maintenance of physically effective fiber to mitigate subacute ruminal acidosis (SARA) risk [20, 21]. While these issues are widely recognized in dairy nutrition, they are rarely discussed in cassava-focused reviews in ways that link residue-specific characteristics, processing-induced changes, and ration level constraints to practical decision rules for inclusion and adaptation. Recent evidence from dairy cows also indicates that responses are context-specific rather than uniformly positive. In multiparous Holstein cows, residue from cassava starch extraction, evaluated at 0, 8, 16, 24, and 32% of the diet dry matter (DM), reduced milk yield by about 15% at the highest inclusion level, indicating that excessive inclusion can compromise productive performance. By contrast, in mid-lactation Thai crossbred cows, replacing soybean meal with fermented cassava pulp with yeast waste did not reduce milk yield or most milk composition traits under the tested conditions, although ruminal ammonia-N increased [22, 23]. These findings underscore why distinctions between high-producing, total mixed ration (TMR)-oriented herds and tropical smallholder or cooperative systems matter: in the former, tight control of fermentability and physically effective fiber is essential for maintaining output and rumen stability, whereas in the latter, feed handling, management capacity, storage conditions, and access to low-cost stabilization are often the binding constraints [9, 10].

Recent advances in bioprocessing research provide a timely foundation for reframing cassava waste utilization in dairy systems from a byproduct-substitution narrative to an upcycling-pathway narrative. Fermentation, ensiling, and other biological treatments can enhance nutritional value and safety, enabling energy and nutrients to be upcycled back into the human food supply via animal production [14]. Consistent with this framing, experimental work on cassava pulp silage demonstrates that combining lactic acid bacteria (LAB) with nitrogen and fermentable substrates (e.g., urea and molasses) can improve fermentation characteristics and aerobic stability, attributes that directly address key barriers to adoption in humid environments [24]. Complementary dairy-focused evidence indicates that fermented cassava pulp products (including fermentation with yeast-related substrates) can be evaluated within lactating cow diets for impacts on rumen fermentation, digestibility, and milk production, supporting the premise that processing is central to converting cassava residues into reliable dairy feed ingredients rather than opportunistic supplements [23]. Accordingly, the transition from simple byproduct substitution to an upcycling pathway should be understood as a sequence of controlled interventions that link stream identity, stabilization route, hazard mitigation, batch verification, and ration-level suitability.

**Despite these developments, there remains a notable gap in the dairy nutrition literature:** a focused synthesis that (i) defines cassava waste streams in a manner consistent with procurement and processing realities, (ii) consolidates the most recent evidence on cyanide risk mitigation and its integration into preservation strategies, and (iii) translates findings into a quality control and feeding framework tailored to lactating cows. More broadly, recent work on circular feed ingredients, such as former foodstuffs, has highlighted that safety and standardization are decisive for acceptance and scaling, with attention to quality assurance and hazard control increasingly central to the scientific and practical discourse [25]. An analogous, residue-specific framework is needed for cassava waste in dairy systems, where both toxicological (HCN-related) and nutritional (rumen stability and milk component) risks must be managed simultaneously. There is also a need to more clearly distinguish between evidence generated in tropical smallholder or cooperative settings and its applicability to high-producing commercial herds, particularly in confinement or TMR-based systems, where tolerance for fermentability errors and effective fiber deficits is lower.

Although cassava processing residues have been investigated as potential ruminant feed ingredients, most studies have examined isolated aspects such as basic nutritional profiles or simple preservation techniques without integrating stream-specific variability, comprehensive hazard control, and dairy-specific ration level outcomes. Safety constraints (cyanogenic glycosides, mycotoxins, microbial hazards) and preservation efficacy are frequently discussed separately rather than as interconnected process-controlled factors. There is also limited evidence linking stabilization methods to rumen function, milk components, and practical implementation in tropical smallholder and cooperative dairy systems, where wet handling and seasonal availability pose unique logistical challenges. This gap limits the translation of cassava residues from opportunistic by-products to reliable circular feed ingredients governed by specification-based procurement and risk management.

Therefore, the objective of this review was to critically evaluate cassava processing residues as circular energy feed ingredients for dairy cows, with particular emphasis on (1) classification and variability of cassava waste streams, (2) cyanogenic glycosides/HCN as a safety constraint and the effectiveness of mitigation strategies, especially ensiling and microbial/chemical interventions, (3) processing pathways that enhance preservation, aerobic stability, and nutritional usability, and (4) ration level considerations for lactating cows, including implications for rumen function and milk production outcomes. The review is oriented primarily toward tropical and subtropical dairy systems, with particular relevance to smallholder and cooperative implementation, while identifying where current evidence remains insufficient for broad generalization to high-producing temperate commercial herds.

### Top of Form

## REVIEW METHODOLOGY

### Top of Form

### Study design

This article was prepared as a structured narrative review to synthesize current evidence on the use of cassava processing residues as circular energy ingredients in dairy systems. The review was designed to integrate evidence across key themes relevant to practical dairy application, including residue stream classification, nutrient composition and variability, safety constraints, preservation and detoxification pathways, and ration level implications for lactating cows. Because the available literature comprises studies with heterogeneous designs, including feeding trials, *in vitro* studies, preservation experiments, and review articles, the evidence was synthesized narratively rather than quantitatively.

### Literature search strategy

A structured literature search was conducted between December 2025 and January 2026 using the electronic databases Scopus, ScienceDirect, and Google Scholar. These sources were selected to capture the literature on cassava processing residues, dairy cow feeding, ruminant nutrition, preservation technologies, and feed safety issues. In addition, the reference lists of key review papers and relevant primary studies were manually screened to identify additional articles directly related to the review's objectives.

### Search terms

The search strategy combined keywords related to cassava residues, ruminant feeding, dairy systems, preservation, and safety. Representative search terms included: “cassava pulp,” “cassava pomace,” “cassava bagasse,” “cassava peel,” “cassava residue,” “cassava waste,” “dairy cow,” “lactating cow,” “ruminant,” “silage,” “ensiling,” “fermentation,” “cyanide,” “hydrogen cyanide,” “mycotoxin,” “aerobic stability,” “feed safety,” “rumen fermentation,” and “milk production.” Search terms were combined using Boolean operators (AND, OR) and were adjusted as appropriate to the indexing and retrieval functions of each database.

### Inclusion and exclusion criteria

The initial search identified 154 records, which were screened based on title and abstract, followed by full-text assessment where necessary. Studies were included if they were published in English and were directly relevant to the use of cassava processing residues in ruminant or dairy feeding systems. Eligible publications were required to provide information on at least one of the following aspects: residue stream identity, nutrient composition, physical characteristics, variability, safety constraints, preservation or detoxification strategies, rumen responses, digestibility, milk production, or milk composition. Studies were excluded if they were not published in English, focused exclusively on cassava for human food use without relevance to residue utilization, feed safety, or animal feeding, lacked sufficient methodological or technical detail, or were duplicate records or otherwise outside the scope of the review.

### Data extraction and synthesis

For each eligible publication, information was extracted on residue type, processing origin, physical form, nutrient composition, physical characteristics, hazard profile, preservation or detoxification method, and reported effects on rumen fermentation, digestibility, milk production, milk composition, or practical feeding value. The retrieved evidence was then organized into thematic sections and synthesized narratively, with emphasis on stream-specific variability, risk-control logic, stabilization strategies, and ration level constraints relevant to lactating cows.

### Scope of the review

This review focuses primarily on cassava processing residues in tropical and subtropical dairy systems, with particular relevance to smallholder and cooperative settings where cassava residues are regionally abundant and wet handling is common. However, the review also considers implications for more intensive and commercial dairy systems where tighter control of fermentability, physically effective fiber, and production responses is required. Evidence from dairy cow studies was prioritized, although selected studies in other ruminants were also included when they provided relevant mechanistic or applied insight into cassava residue utilization, preservation, or safety.

## DEFINING CASSAVA WASTE STREAMS FOR DAIRY FEEDING

A stream-based taxonomy is a prerequisite for credible synthesis and practical adoption because cassava waste is routinely used as an umbrella term for materials that differ markedly in residual starch vs. fiber, cyanogenic potential, and handling stability. Recent work on cassava as a ruminant feedstuff emphasizes the need to connect feeding recommendations to specific residue types and processing contexts, rather than treating cassava residues as nutritionally interchangeable [4].

## PROCESSING PATHWAYS AND WHERE EACH RESIDUE ORIGINATES

Cassava residues entering dairy feed systems are generated at distinct steps of root processing. Industrial starch extraction typically involves washing and peeling (yielding peels/rinds), size reduction/rasping, and starch separation (yielding a solid fibrous fraction, pulp/pomace/press cake, plus effluents), followed by subsequent dewatering/drying or downstream valorization [26–28]. A recent overview of cassava biorefineries highlights that cassava industry residues include fibrous by-products such as bagasse (scraped pulp and peel) and large volumes of liquid streams, reflecting how processing configurations shape the residue portfolio and contamination risks (e.g., soil/ash, dilution by wash water) [29]. This matters for dairy feeding because the same pulp label can reflect different separation efficiencies: higher starch recovery generally produces a more fibrous pulp with lower residual starch, whereas lower recovery yields pulp with higher residual starch and greater fermentability. As a practical benchmark, dried cassava pulp has been reported to contain approximately 42.8–64.0% starch on a DM basis, with an average near 53.4% DM; thus, pulp from more efficient industrial recovery may fall toward the low-40% range, whereas pulp from less efficient recovery may remain in the high-50% to about 60% DM range [30, 31]. Recent ruminant-focused syntheses similarly argue that feeding strategies must be tailored to how cassava is processed in specific regions and supply chains [4].

## PHYSICAL FORMS (FRESH/WET, DRIED, PELLETED, ENSILED, FERMENTED) AND LOGISTICS IMPLICATIONS

The feasibility of cassava residues in dairy rations is strongly determined by physical form and logistics. Fresh/wet pulp (often high moisture) is typically highly perishable in warm climates and therefore requires rapid stabilization (e.g., ensiling) or near-immediate feeding; otherwise, aerobic spoilage and nutrient losses can be substantial. Processing studies on cassava pulp silage show that combining LAB with fermentable substrate and nitrogen sources (e.g., molasses and urea) can improve fermentation profiles and aerobic stability, directly addressing a key adoption bottleneck for wet residues [24, 32–34]. Complementary recent work evaluating treated cassava pulp demonstrates that such interventions can also modify *in vitro* gas kinetics and rumen fermentation characteristics, supporting the view that stabilization is not merely preservation but can change feeding value [35]. Dried or pelleted residues reduce spoilage and transport costs per unit of DM [36–39] but introduce processing costs and may concentrate ash/soil contamination if present [40–42]; they also shift risk toward storage quality control (e.g., re-wetting, mold). Ensiled/fermented forms can be advantageous for cooperatives and smallholders by enabling bulk seasonal storage [43, 44], but require specification-based management (target DM, packing density, fermentation time, and aerobic stability after opening). More broadly, recent ensiling research involving cassava-derived materials emphasizes that additive choice and microbial ecology can be leveraged to improve fermentation performance, an insight directly transferable to cassava residue stabilization strategies [32].

## WHY STREAM IDENTITY MATTERS

**Stream identity is consequential for two reasons:** nutrition and hazards. Nutritionally, cassava-derived feed ingredients are generally characterized as high-starch, low crude protein, low-fat materials, but the extent of residual starch and the fiber fraction can differ substantially by stream and processing [45–47]. Hazard-wise, cassava tissues contain cyanogenic glycosides (notably linamarin and lotaustralin) that may release HCN; peels and foliage streams tend to be more hazard-sensitive than many pulp streams, and mechanical disruption plus moisture can increase cyanide release [48, 49]. Recent systematic synthesis on cassava product safety reinforces the importance of expressing HCN consistently (e.g., mg/kg) and benchmarking against guideline thresholds, which is equally relevant when translating safety logic to animal feed supply chains [50]. Moreover, high moisture solids such as wet cassava pulp deteriorate rapidly, and this deterioration not only reduces nutritive value but also promotes the proliferation of yeasts, molds, and other spoilage organisms, potentially increasing heating, intake depression, and the risk of mycotoxin formation if drying or ensiling is inadequate. By contrast, dried or pelleted forms reduce microbial spoilage pressure but require control of ash contamination, re-wetting, and post-processing moisture ingress [7, 51, 52]. Together, these considerations justify a taxonomy that explicitly links each residue stream to (i) its origin and processing, (ii) its physical form at feeding, and (iii) its hazard control requirements, enabling more defensible cross-study comparisons and more actionable dairy feeding guidance (Table 1) [4, 53–56].

**Table 1:** Stream-based taxonomy of cassava processing residues for dairy feeding.

**Residue stream**	**Origin in processing chain**	**Typical physical forms**	**Main nutritional features**	**Main hazards**	**Preferred stabilization route**	**Recommended analytical checks**	**Typical relevance in dairy feeding**	**Reference**
Cassava pulp / pomace	Solid fraction remaining after starch/flour extraction and dewatering/pressing [31]	Wet bulk, dried, pelleted, ensiled, fermented [24]	Variable residual starch and fiber; low CP; mainly an energy ingredient	Rapid spoilage when wet; batch variability; ash/soil contamination; Mold risk if poorly dried or air-exposed [45]	Rapid ensiling, drying, or controlled fermentation	DM, starch, aNDFom/NDF, ash, acid-insoluble ash, total cyanide, visible spoilage, pH/temperature after opening	Main candidate for partial cereal replacement when quality is controlled	[4, 53]
Cassava peels / rind	Generated during peeling after washing/cleaning [29]	Fresh; sun-dried; meal; ensiled	More fibrous and usually lower-energy than pulp; variable with peeling efficiency	Higher cyanogenic risk; variable ash/soil; palatability constraints; spoilage when wet [54]	Drying or ensiling after size reduction; often mixed with other materials	DM, NDF, ash, acid-insoluble ash, total cyanide, visible contamination	Energy/fiber contributor at controlled inclusion where detoxification and QC are available	[53, 54]
Cassava foliage/tops	Harvest residues or dedicated forage streams [55]	Fresh forage; chopped; dried leaf meal, mixed silage	Fiber plus variable protein contribution; maturity-dependent	Cyanogenic glycosides; maturity-related variability; fermentation inconsistency [55]	Co-ensiling with grasses or drying	DM, CP, NDF, total cyanide, fermentation profile if ensiled	More relevant as co-ensiled forage than as a stand-alone concentrate substitute	[4, 54]
Mixed pulp-peel streams	Mixed solids resulting from factory configuration or incomplete separation [29]	Wet bulk; dried; ensiled	Highly variable starch: Fiber ratio; unpredictable energy value	Combined hazard profile: ash contamination, cyanogenic risk and spoilage; inconsistent nutritive value unless specification-based procurement used [29]	Specification-based procurement followed by ensiling or drying	DM, starch, NDF, ash, acid-insoluble ash, total cyanide	Opportunistic energy/fiber source where consistent supply and QC exist	[4, 53]
Cassava residue / tapioca residue	Post-extraction solids variably defined across datasets and markets [45]	Typically dried/pelleted; sometimes wet locally	Composition uncertain because stream identity is unclear	Definition ambiguity; poor cross-study comparability; unknown hazard profile if origin is not specified [45]	Use only when product specifications are available	Explicit reporting of origin, form, DM, starch, fiber, ash, and cyanide where relevant	Can be used in concentrate only when analytically defined	[4, 53]
Wastewater / effluent-derived solids	Recovered from washing, screening, sedimentation, or wastewater handling	Wet sludge/solids, sometimes sun-dried or mixed before ensiling	Highly variable; may contain fine starch-rich particles but also high moisture and contaminants	Very poor stability; microbial contamination risk; possible inorganic contamination; difficult handling	Dewatering plus drying or controlled co-ensiling if used	DM, ash, acid-insoluble ash, microbiological quality, total cyanide when relevant	Use with caution; generally lower priority unless recovery is standardized	[55, 56]
Bioethanol or modified starch residues	Residues from bioethanol production, modified starch, or downstream industrial processing	Wet co-products, dried residues, mixed industrial solids	Composition depends strongly on process; not directly comparable with conventional pulp	Definition ambiguity; variable fermentability; uncertain hazard profile without process disclosure	Case-specific; requires full specification before feed use	Process description, DM, starch/sugars, NDF, ash, cyanide where relevant	Emerging streams with potential, but unsuitable for generalization without specification	[4, 56]

DM = Dry matter, CP = Crude protein, NDF = Neutral detergent fiber, aNDFom = Neutral detergent fiber assayed with a heat-stable amylase and expressed exclusive of residual ash (ash-free NDF), QC = Quality control.

**NUTRIENT COMPOSITION, PHYSICAL CHARACTERISTICS, AND VARIABILITY** **TYPICAL NUTRIENT CHARACTERISTICS RELEVANT TO LACTATING COWS**

Across cassava waste streams intended for ruminant feeding, the dominant nutritional attribute is a high supply of fermentable carbohydrates (residual starch plus rapidly fermentable non-starch polysaccharides), coupled with consistently low crude protein (CP) and ether extract, making these materials primarily energy carriers rather than protein sources [22, 57–64]. Dried cassava pulp/pomace (dehydrated starch residue) typically contains substantial starch (reported roughly in the 43–64% DM range) but has highly variable neutral detergent fiber (NDF) (up to ~40%+ DM), reflecting heterogeneous separation efficiency and fiber carryover during starch extraction [30, 31, 65–69]. Cassava residue from flour/starch processing can shift further toward a mixed starch–fiber profile, with starch commonly remaining near the mid-40% range while total dietary fiber may approach 35% [70, 71], reinforcing that cassava waste is not a uniform ingredient class. In practical dairy formulation, minerals are usually not a primary benefit of cassava residues (phosphorus can be low in several cassava-derived ingredients), but ash should be interpreted together with acid-insoluble ash (AIA), because total ash includes intrinsic plant minerals whereas AIA is a more specific indicator of soil or sand contamination that can dilute energy density and increase equipment wear [31, 72]. To complement the narrative discussion of compositional variability, Table 2 summarizes recent literature indicating that cassava-derived feed resources vary not only in nutrient concentrations but also in functionally important attributes related to processing history, safety, and practical feeding value [22, 24, 54].

## PHYSICAL CHARACTERISTICS

Physical characteristics are equally decisive for lactating cows because they govern intake dynamics, mixing behavior, sorting risk, and ruminal buffering [9, 73–75]. Wet cassava pulp/pomace is commonly delivered at very low DM (often near 13–17% as-fed depending on source and drainage), which can constrain inclusion rates through bulk, transport cost per unit DM, and rapid spoilage pressure [76], while dried or pelleted forms reduce logistics constraints but can change eating rate and effective fiber contribution [77–79]. Processing history should be considered alongside chemical composition because cassava pulp with similar starch concentrations may differ markedly in functional feeding value. Heat drying can alter the physicochemical properties of starch, including gelatinization-related behavior and granule accessibility, whereas ensiling or fermentation can modify the surrounding matrix and pre-digest part of the substrate. Consequently, fresh, dried, ensiled, or fermented cassava pulp with comparable starch concentrations may differ in ruminal starch digestibility and therefore in predicted net energy for lactation (NEL). This interpretation is consistent with recent work showing that additive-treated ensiled cassava pulp can alter gas kinetics, rumen fermentation characteristics, and degradability, even when the material remains recognizably a cassava pulp residue [35, 80]. Ensiled/fermented forms sit between these extremes: they preserve wet material and can stabilize handling, but their nutritional interpretation must explicitly account for additives (e.g., urea or molasses) and fermentation outcomes, because these can materially alter equivalent CP (via N addition), fiber fractions, and ruminal nitrogen supply [81, 82].

## REVISED DRIVERS OF VARIABILITY AND HAZARDS AND SAFETY CONSTRAINTS

### Drivers of variability

The largest driver of variability in cassava pulp/pomace is the processing plant’s starch recovery efficiency. Even modest shifts in screening, rasping, washing intensity, and separator performance can redistribute carbohydrate between the starch product and the residue, producing large swings in residual starch versus structural carbohydrate (and therefore metabolizable energy/NEL potential and rumen fermentation kinetics) [30]. Dilution and wash water carryover further contribute to inconsistency by altering as-fed DM and concentrating or diluting soluble fractions, which in turn affect the delivered nutrient density and the actual inclusion rate in a TMR on an as-fed basis, an error mode that is disproportionately important for wet streams [83–87]. Soil contamination and inadequate cleaning/peeling increase ash (particularly insoluble ash), depressing energy density and potentially introducing undesirable inorganic load; this is especially relevant for residues handled on the ground or dried under conditions that promote contamination.

**Table 2:** Recent literature summary of nutrient variability and functionally relevant attributes of cassava-derived feed resources.

**Feed resource / stream**	**Processing condition**	**Key composition data (DM basis unless stated)**	Mean / range / CV*	**Dairy relevance**
Cassava pulp	Wet pulp before fermentation	DM 165.5 g/kg as-fed; CP 25.94 g/kg DM; NDF 438.87 g/kg DM; ADF 246.79 g/kg DM	Single-source baseline; CV not reported	Very wet material with high fiber and low CP; useful baseline for interpreting spoilage risk, dilution of nutrient density, and the need for rapid stabilization [24]
Cassava pulp silage	21-d fermentation with *L. casei* TH14, urea, and/or molasses (8 treatment combinations)	DM 166.10–177.62 g/kg as-fed; CP 21.34–154.69 g/kg DM; NDF 334.71–394.37 g/kg DM; ADF 198.84–222.94 g/kg DM	Mean DM 171.65 g/kg, CV 2.7%; mean CP 80.66 g/kg DM, CV 77.8%; mean NDF 351.35 g/kg DM, CV 5.5%	Shows that fermentation additives can dramatically change apparent CP and modestly shift fiber fractions even within the same cassava pulp stream; supports using equivalent crude protein / NPN language and not relying on starch or CP alone to predict NEL. Aerobic stability was >120 h after silo opening across additive treatments [24]
Cassava peel	Fresh peel vs hot-water control peel vs enzyme-treated peel	Fresh peel NDF 61.01% DM, ADF 57.00% DM, CF 35.01% DM, cyanide 308.35 μg/g; control peel NDF 35.13% DM, ADF 31.55% DM, cyanide 281.75 μg/g; enzyme-treated peel NDF 33.16–33.18% DM, ADF 24.01–28.91% DM, cyanide 126.61–142.17 μg/g	Clear processing-driven range rather than pooled mean/CV	Strong example of how processing changes both safety and fiber burden; peels are not nutritionally interchangeable with pulp and should be treated as a more fibrous, more cyanide-sensitive stream [54]
REAM (residue from extraction of cassava starch)	Included in diets of multiparous Holstein cows	Inclusion levels 0, 8, 16, 24, and 32% of diet DM	Performance range rather than composition range	At 32% of diet DM, milk yield was reduced by about 15%; authors concluded that up to 16% of diet DM could be included, whereas higher levels reduced productivity. This is a useful recent anchor linking residue variability and ration fit to biologically meaningful dairy outcomes [22]

*Values are reported from recent studies most directly relevant to cassava-derived feed resources. CVs are shown only when they can be calculated from multiple treatment means reported in a recent study; otherwise, mean or range values are presented as available. DM = Dry matter, CP = Crude protein, NDF = Neutral detergent fiber, ADF = Acid detergent fiber, CF = Crude fiber, CV = Coefficient of variation, NPN = Non-protein nitrogen, NEL = Net energy for lactation, REAM = Residue from extraction of cassava starch. Unless otherwise indicated, nutrient composition is expressed on a DM basis.

**Upstream agricultural variation also matters:** cultivar, agronomic conditions, and season influence root composition (starch, fiber, and cyanogenic potential), and these differences propagate into waste streams, particularly when factories draw from mixed-cultivar pools across seasons [88–92]. Finally, post-processing choices (draining time, sun-drying vs. mechanical drying, pelletizing, ensiling inoculants, urea/molasses use) can shift both apparent nutrient composition and functional value; for example, urea addition predictably elevates analyzed CP in cassava pulp silage largely as non-protein nitrogen (NPN), creating a product that is analytically higher protein but biologically still distinct from true-protein feeds [24].

**How variability translates to ration uncertainty (fermentability, effective** **fiber** **, milk fat risk)**

For dairy cows, the practical consequence of compositional spread is ration uncertainty in three linked domains: (i) ruminal fermentability (rate and extent of starch/fiber digestion), (ii) physically effective fiber (chewing stimulation and rumen pH stability), and (iii) downstream risks of SARA and milk fat depression (MFD). When cassava pulp/pomace arrives with higher-than-expected starch and lower effective fiber contribution, it can behave more like a rapidly fermentable concentrate, increasing acid load and compromising rumen pH unless physically effective NDF (peNDF) and buffering capacity are protected at the ration level [93]. This matters because the milk fat response is sensitive not only to how much starch is available but also to how fast and where it ferments, interacting with the unsaturated lipid supply and rumen biohydrogenation pathways that produce MFD-associated fatty acid intermediates [94]. Conversely, when NDF in cassava pulp/residue is elevated (or particle characteristics change with drying/pelleting), the ingredient may contribute more to fiber supply but not necessarily to physically effective fiber unless particle size distribution and fragility are known, an issue increasingly emphasized in modern dairy feed characterization frameworks [95]. Recent *in vitro* work with ensiled cassava pulp further illustrates that processing and additive choices can alter digestibility and fermentation traits, meaning that cassava pulp silage is not one feed but a family of feeds whose risk profile depends on how it was produced and how consistently it is described analytically [35]. The contribution of cassava pulp to physically effective fiber should be interpreted cautiously, because NDF concentration alone does not ensure adequate peNDF if particles are small, fragile, or further reduced during TMR mixing. More broadly, dairy evidence shows that peNDF influences chewing activity, rumen fermentation, plasma metabolites, and milk production in high-concentrate diets, supporting the need to evaluate cassava residues in the context of whole-ration particle size and fermentability rather than as isolated chemical ingredients. In practical terms, variability in starch accessibility, particle characteristics, and contamination burden should be expected to affect not only estimated energy value but also rumen pH stability, volatile fatty acid pattern, chewing response, and ultimately milk component responses when cassava residues are incorporated into lactating cow diets. Recent dairy cow evidence also suggests that these uncertainties are biologically meaningful: residue from cassava starch extraction included at 0, 8, 16, 24, and 32% of diet DM reduced milk yield at the highest inclusion level, linking compositional and physical variability more directly to performance risk [22, 23].

### Hazards and safety constraints

The safe use of cassava processing residues in dairy systems depends on recognizing that hazards arise from both intrinsic plant chemistry (cyanogenic glycosides), and extrinsic contamination and preservation failures (fungal toxins, pathogenic microorganisms, inorganic contaminants, and chemical residues). Because high-producing lactating cows are routinely fed high-concentrate diets and large daily DM intakes, even moderate deviations in hazard load or preservation quality can translate into clinically relevant outcomes, reduced milk performance, or milk safety concerns. A hazard-focused synthesis is therefore essential to move cassava residues from opportunistic by-products to reliable circular feed ingredients governed by specification-based procurement and process control.

**CYANOGENIC GLYCOSIDES AND HCN:** A RESIDUE-SPECIFIC TOXICOLOGICAL CONSTRAINT

Cassava tissues contain cyanogenic glycosides (primarily linamarin and lotaustralin) that can be converted to HCN when plant cells are disrupted and enzymatic hydrolysis proceeds, processes that are intensified by chopping, grinding, or maceration and by moist conditions [54]. From a dairy feeding perspective, the risk is stream-dependent: peels- and foliage-derived streams are typically a greater concern than many starch extraction pulps, and the risk increases when fresh materials are fed soon after processing (i.e., before detoxification via drying or fermentation) [54]. In ruminants, low-level cyanide exposure can be detoxified primarily by conversion to thiocyanate [96–100], but rapid absorption of large amounts can overwhelm detoxification capacity and lead to acute poisoning; rumen conditions (including pH) influence the rate and extent of cyanide release [101]. These mechanistic considerations matter operationally because they imply that safe inclusion cannot be defined by a single percentage of diet DM; it must account for stream identity, processing state, and the rate at which cyanide can be released and absorbed.

Recent research has progressed from descriptive hazard recognition to practical mitigation strategies directly relevant to dairy supply chains. Ensiling is particularly important because it can simultaneously address preservation and detoxification, and recent studies demonstrate that inoculation strategies can alter residual cyanide outcomes. A study evaluated cassava root silage inoculated with cyanide-utilizing bacteria isolated from the bovine rumen and observed measurable reductions in total cyanide, along with changes in fermentation quality and *in vitro* rumen fermentation indices [15]. Complementary evidence indicates that supplementing cattle receiving fresh cassava root with cyanide-utilizing bacteria and sulfur can improve cyanide degradation and modify rumen fermentation and microbiome profiles [102], supporting a plausible biological basis for integrating targeted microbial and nutritional interventions into cassava-feeding programs. More broadly, a recent applied review on cassava byproduct valorization emphasizes that detoxification is a prerequisite for wider utilization of peels and leaves, reinforcing the need for consistent hazard control rather than ad hoc feeding decisions [54].

**From a dairy feeding perspective, HCN risk is stream- and process-dependent:** peels- and foliage-derived streams are generally of greater concern than many starch extraction pulps, and risk increases when fresh materials are fed before being dried or fermented. Although high-producing dairy cow-specific residual HCN thresholds remain insufficiently standardized, published lactation studies suggest that dietary exposures of 35–70 ppm HCN from fresh cassava pulp and approximately 75 ppm HCN from fresh cassava peel can be tolerated under the reported study conditions without adverse effects on milk yield or milk composition. These values, however, should not be interpreted as universal safety limits for high-producing herds, because cyanide risk depends on stream identity, intake rate, sulfur status, rumen conditions, and the speed of cyanide release and absorption. Recent ensiling work also shows that detoxification efficacy remains incomplete and process-sensitive: after 30 days of ensiling, cyanide removal from cassava root silage was about 39% in untreated silage and improved to approximately 47–51% with cyanide-utilizing inoculants and/or cellulase, indicating that longer-term efficacy and the interaction with rumen microbial adaptation remain important research gaps rather than settled control measures. Low-level cyanide exposure is detoxified primarily to thiocyanate, and dairy studies show that milk thiocyanate can increase when fresh cassava materials are fed; for example, milk thiocyanate concentrations of 1.92–14.58 mg/dL have been reported in cows fed fresh cassava root. Accordingly, HCN should be managed as a process-controlled hazard, with preference for documented detoxification routes, conservative use of fresh high-cyanogen streams in high-producing cows, and residual cyanide testing where feasible [15, 103–105].

**MYCOTOXINS AND FUNGAL SPOILAGE:** HAZARDS AMPLIFIED BY WET RESIDUES AND POOR DRYING/ENSILING

Cassava residues, particularly when handled as wet pulps or inadequately dried products, can be vulnerable to fungal growth and mycotoxin contamination, especially in humid tropical conditions and in supply chains with prolonged storage, intermittent drying, or re-wetting [51, 52, 106–108]. Cassava-specific survey data indicate that this is not merely a theoretical concern. In a recent value-chain study in Uganda, 192 cassava product samples (flour and chips) were screened for multiple mycotoxins, and the detected profile included aflatoxins, fumonisins, ochratoxin A, deoxynivalenol, zearalenone, and citrinin; all positive samples exceeded the EU threshold of 5 μg/kg for Aflatoxin B1 (AFB1). These findings support the view that cassava matrixes may harbor multi-mycotoxin contamination, not just isolated aflatoxin events, and therefore require a broader surveillance approach than single-toxin screening alone. This is especially relevant for dairy systems because aflatoxin B1 in feed can be converted to aflatoxin M1 (AFM1) in milk. Recent review evidence indicates that AFB1 carryover into milk is typically about 1–2% on average but can reach approximately 6% in high-yielding cows, meaning that cows may show no obvious clinical signs while milk still exceeds regulatory limits. More broadly, a recent meta-analysis of bovine feeds indicates that aflatoxins are especially prevalent in warmer, drier climates and that climate change may shift current and emerging mycotoxin risks, which strengthens the case for routine surveillance when cassava residues are introduced into lactation diets [109–111]. A major step toward standardizing prevention is the Codex Code of Practice (CXC 82-2023) for the prevention and reduction of mycotoxins in cassava and cassava-based products, which explicitly recognizes that toxigenic fungi are associated with soil/dust, crop residues, and post-harvest storage/processing environments, and that risk profiles vary by region and handling practices [112]. Although this Codex guidance is written primarily for food chains, the same hazard logic applies to feed chains, as cassava residues often share upstream storage and processing nodes.

Recent empirical work underscores the relevance of multi-mycotoxin contamination along cassava value chains. A study reported the presence of multiple regulated and emerging mycotoxins (including aflatoxins, fumonisins, ochratoxin A, deoxynivalenol, zearalenone, citrinin) in cassava products sampled across a national value-chain, illustrating that cassava matrixes can exhibit complex co-contamination patterns rather than single-toxin profiles [109]. From the dairy nutrition angle, this matters because lactating cows are sensitive to chronic mycotoxin exposure through impacts on intake, immune function, reproduction, and milk production, and because aflatoxin B1 in feed is a well-recognized precursor to AFM1 in milk (risk magnitude depends on exposure and carryover factors) [110, 113, 114]. A recent environmental health synthesis on mycotoxins in bovine feed highlights that mycotoxin exposure remains widespread across feed components and that climate variability may shift prevalence, underscoring the need for routine surveillance and risk-based testing strategies [111]. Climate variability and warming trends should also be considered, as recent global evidence indicates that aflatoxin risk is favored by warmer, drier conditions, implying that cassava-based feed chains in tropical regions may face increasing contamination pressure unless drying, storage, and surveillance are improved [111]. Practical guidance for livestock producers emphasizes that risk depends on toxin concentration, intake rate, animal class, and exposure duration, conditions that are directly relevant when introducing variable by-products into high intake lactation diets [115].

Furthermore, fresh or insufficiently stabilized cassava pulp should be treated as highly vulnerable to aerobic deterioration. Once exposed to air, yeasts initiate oxidation of residual sugars and fermentation acids, which manifests as heating, rising pH, and loss of palatability before visible mold becomes apparent. In practical terms, heating of the pulp pile should be interpreted as an early warning sign of yeast-driven deterioration rather than a benign temperature fluctuation, because pH elevation and acid depletion create conditions that favor broader spoilage microbiota and, over time, mold proliferation and hygiene failure. For field monitoring, aerobic stability is commonly defined as the time until silage temperature rises 2°C above ambient. In cassava pulp silage studies, additive-treated silages have been reported to maintain>120 h of aerobic stability after opening, whereas cassava residue silages with pH below 4.0 are generally described as well preserved. These values are not universal guarantees, but they provide practical benchmarks for quality control in cooperatives and smallholder systems [24, 116, 117].

## IMPLICATIONS FOR DAIRY PRACTICE

Cassava residues should be incorporated into farm-level mycotoxin management plans with (i) specification-based procurement (moisture limits, visible mold rejection, storage conditions), (ii) periodic screening (rapid tests for aflatoxins where relevant; Liquid chromatography-tandem mass spectrometrywhen diagnosing complex issues), and (iii) documented storage and stabilization methods that minimize aerobic spoilage and fungal growth.

**MICROBIOLOGICAL HAZARDS IN ENSILED/FERMENTED FORMS:** *CLOSTRIDIA*, *LISTERIA*, AND HYGIENE FAILURES

When cassava pulp is stabilized via ensiling, the hazard profile shifts from rapid aerobic spoilage toward risks associated with suboptimal fermentation and pathogen persistence, particularly if packing density is poor, DM is too low, or oxygen ingress occurs during storage and feed-out. A recent review on silage pathogens and biological control agents highlights that silage fermentation can be compromised by undesirable microorganisms, including yeasts, molds, Clostridia, and *Listeria* spp., with consequences for animal performance and feed hygiene [118]. Dairy-relevant evidence indicates that poor-quality silage is a major risk factor for *Listeria monocytogenes* on farms, and that *Listeria* can persist in farm environments even with good hygiene, emphasizing the centrality of silage quality and feed-out management [119]. A mechanistic review of LAB performance in silage production further clarifies why fermentation success is not guaranteed: silage outcomes depend on substrate composition, epiphytic microbiota, moisture, compaction, and additive choice, variables that are especially volatile in non-forage residues like wet cassava pulp [120].

In dairy practice, if cassava pulp is ensiled, safety constraints should include minimum DM targets, rapid sealing, sufficient packing density, and management of aerobic stability at feed-out. Inoculant selection should be justified by expected substrate limitations (e.g., sugar availability, buffering capacity) and the specific goal (preservation, improved aerobic stability, or detoxification support) [120].

**INORGANIC AND CHEMICAL CONTAMINANTS:** SOIL/ASH, HEAVY METALS, AND PESTICIDE RESIDUES

Beyond biological hazards, cassava residues can carry inorganic contaminants introduced via soil adhesion, drying on bare ground, or processing environments. Elevated ash, particularly insoluble ash, reduces energy density and can indicate contamination that may co-occur with trace metals depending on local soil conditions. Recent research on tuber crops reports widespread attention to heavy metal contamination (e.g., Pb, Cd, As, Hg) at a population-sample level, suggesting a plausible risk pathway for cassava-derived materials in contaminated environments [121]. While many heavy metal studies focus on food, the same agronomic drivers apply to feed chains, and a precautionary approach is warranted when residues are sourced from regions with known industrial or mining activity in soils.

Chemical residues (e.g., pesticides) are another emerging consideration, not because cassava is uniquely pesticide-intensive, but because feed chains increasingly detect diverse residues across plant-based feedstuffs. A 2019–2023 monitoring study using gas chromatography-tandem mass spectrometry found pesticide residues in a high proportion of feed samples (region-specific), illustrating that multi-residue contamination is common in modern feed systems and can shift over time [122]. Regulatory frameworks (e.g., EU coordinated control programs for pesticide residues in food/feed commodities) reinforce that residue monitoring is a continuing policy priority and that feed and food chains are analytically linked [123].

In dairy practice, chemical and inorganic hazards should be managed through supplier qualification (source-area risk screening), ash/insoluble ash as a routine indicator for soil contamination, and targeted testing when sourcing from high-risk regions or when unexplained performance/health issues occur.

## AN IMPLEMENTATION-ORIENTED HAZARD CONTROL LOGIC FOR DAIRY SYSTEMS (MINIMUM REQUIREMENTS)

**Taken together, these hazards argue for a practical, tiered safety framework:** (i) stream identification (pulp vs. peel vs. mixed) with declared origin and physical form; (ii) process documentation (drying parameters, ensiling protocol, inoculants/additives, storage duration); (iii) batch acceptance criteria (moisture/DM, visible spoilage, ash/soil indicators; cyanide indicators and/or testing where feasible); and (iv) monitoring during use (silage heating, aerobic spoilage, intake disruption, milk fat changes, and health signals). This logic aligns with modern thinking in circular feed valorization, namely that residues become scalable feed ingredients only when they are governed by measurable specifications and controlled processes rather than by informal opportunistic use.

**UPCYCLING PATHWAYS:** PRESERVATION AND DETOXIFICATION AS ENABLING TECHNOLOGIES

Upcycling in the cassava residue context is not synonymous with simply using a byproduct; it refers to a sequence of enabling technologies that (i) stabilize a highly perishable, high moisture substrate, (ii) reduce cyanide (HCN) risk where relevant, and (iii) yield a feed ingredient with predictable nutritive value and handling characteristics suitable for high intake lactating cows. The practical implication for dairy cooperatives is that processing should be framed as a decision system: select the route (drying vs. ensiling vs. fermentation/bioconversion) based on incoming moisture, cleanliness (ash/soil), spoilage status, and hazard profile (Table 3) [15, 24, 35, 96, 124–128]. Because these pathways differ in detoxification efficiency, DM losses, labor demand, aerobic stability, and final feeding value, they should be compared not only by processing name but also by stream suitability, quantitative outcomes, and likely failure modes under field conditions.

### Drying

Drying converts a wet residue stream into a storage-stable commodity by lowering water activity and limiting microbial growth, often improving marketability and transport efficiency per unit of DM. However, drying is best understood as a trade-off between stability gains and energy/capital costs, with additional risk management needs (e.g., contamination during sun-drying and moisture re-absorption during storage). From a detoxification perspective, multiple recent studies in cassava matrixes show that drying temperature and duration can measurably reduce cyanide-related compounds, although the magnitude depends strongly on the cassava tissue type and process conditions [129, 130]. Importantly, drying should not be interpreted as a uniform detoxification process: cyanide reduction requires time for endogenous linamarase to hydrolyze cyanogenic compounds before volatilization occurs, so slower sun-drying or staged drying may, in some cases, reduce cyanogens more effectively than very rapid, high-temperature drying. Conversely, flash or very rapid drying may improve storage stability but shorten the hydrolysis window, thereby reducing detoxification efficiency. For example, controlled drying experiments on cassava leaves show faster drying rates at higher temperatures and link drying conditions to quality outcomes, illustrating why drying must be specified by method and time–temperature profile rather than treated as a uniform intervention [131]. Complementary drying-process studies on cassava chips similarly report reductions in moisture and cyanide metrics with longer drying durations, supporting the general principle that cyanide mitigation via drying is process-sensitive and should be validated for the specific stream and equipment used [132]. For dairy applications, drying is often most defensible when (i) cooperative logistics cannot reliably support rapid ensiling, (ii) the residue is clean enough to avoid ash concentration, and (iii) the economic case (fuel/solar infrastructure, labor, and shrink losses) is favorable relative to ensiling. Accordingly, drying decisions should balance storage stability against detoxification performance rather than assuming that faster moisture removal automatically produces the safest feed ingredient.

### Ensiling

Ensiling is frequently the most practical upcycling route for cassava pulp because it simultaneously provides preservation and, in certain cassava tissues, reduces cyanide via fermentation-driven biochemical changes. Recent cassava silage studies demonstrate that inoculation strategies and enzyme additions can influence both fermentation quality (e.g., lactic acid production, pH decline) and cyanide outcomes. A controlled experiment on fresh cassava roots showed that ensiling reduced total cyanide, and that adding cyanide-utilizing bacterial inoculants (with or without cellulase) further improved cyanide reduction relative to an untreated control, while maintaining acceptable silage quality metrics [15]. In cassava pulp systems specifically (a high moisture, low-protein residue), fermentation is also a tool to increase the functional value of the ingredient by improving preservation and reducing losses. A study using *Lacticaseibacillus* (*Lactobacillus*) *casei* TH14 with urea and molasses documented improved silage fermentation end products and reported markedly enhanced aerobic stability under certain additive combinations, highlighting that silage success depends on matching the additive strategy to substrate limitations [24]. Beyond cassava, broader syntheses of silage microbiology emphasize that fermentation trajectories depend on substrate composition, epiphytic microbiota, packing density, and additive choice—factors that can be especially variable in byproduct silages [120].

**Table 3:** Processing methods and outcomes.

**Processing pathway**	**Best-suited stream/form**	**Primary objective**	**Quantitative outcomes reported**	**Main limitations / failure risks**	**Practical dairy interpretation**	**Reference**
Drying (sun/mechanical)	Peels, pulp, mixed wet solids in sunny conditions	Moisture reduction, storage stabilization, partial detoxification	Reduces moisture sufficiently for storage; cyanide reduction may be greater than under very rapid drying when linamarase has time to hydrolyze cyanogenic compounds before volatilization	Weather dependence, contamination during open-air drying, labor demand, variable final moisture	Low-cost option for smallholders/cooperatives where climate permits; should not be assumed equivalent to rapid industrial drying for detoxification	[124]
Rapid hot air	Industrial pulp/bagasse streams	Fast moisture reduction and transportability	Strong moisture removal, but very rapid drying may shorten the hydrolysis window needed for efficient cyanide reduction	Fuel/equipment cost; residual cyanide may remain if detoxification is assumed rather than verified	Suitable for logistics and shelf life, but cyanide control should be analytically verified	[124, 125]
Ensiling without additives	Wet cassava pulp or root-rich streams	Preservation under anaerobic fermentation; partial detoxification	In cassava root silage, cyanide removal after 30 d was about 39% in untreated silage	Fermentation failure if packing/sealing is poor; variable post-opening stability	Practical baseline option for wet materials near source, but not a complete detoxification guarantee	[15]
Ensiling + LAB inoculant	Wet cassava pulp, mixed silages	Improve fermentation consistency and suppress undesirable microbes	Faster pH decline; improved fermentation profile; in cassava pulp silage studies, aerobic stability may exceed 120 h after opening under experimental conditions	Requires inoculant access and mixing consistency; response depends on substrate and epiphytic flora	Strong option when wet material must be preserved quickly and QC is feasible	[24, 35]
Ensiling + molasses	Wet pulp, mixed silages, foliage-grass mixtures	Increase fermentable substrate and support lactic fermentation	Improved acidification and fermentation profile where fermentable sugar is limiting	Added cost; over-reliance may not solve poor compaction or air ingress	Useful support additive, especially in mixed or low-sugar silages	[126]
Ensiling + urea (± molasses, ± LAB)	Wet cassava pulp	Improve preservation and increase rumen-available N	Apparent CP rises substantially, but this is mainly NPN / equivalent crude protein, not true-protein; additive combinations also improve aerobic stability	Uneven mixing, ammonia odor, palatability issues, confusion between CP and true-protein enrichment	Useful where rumen N supply is limiting, but should be reported as NPN/equivalent CP	[35]
Ensiling + cyanide-utilizing bacteria (± cellulase)	Cyanogen-sensitive root or peel-rich wet streams	Detoxification plus preservation	Cyanide removal improved from about 39% in untreated silage to about 47–51% with inoculants and/or activated carbon after 30 d	Inoculum availability, cost, scale-up uncertainty, incomplete detoxification	Promising targeted approach where HCN risk is high, but still requires validation and residual testing	[96]
Co-ensiling / mixed-substrate silage	Foliage + grass, wet pulp + drier by-products	Balance moisture, improve fermentation, moderate nutrient profile	Can improve fermentation profile and handling where single-stream moisture is excessive	Formulation inconsistency; variable nutrient composition between batches	Particularly useful in smallholder/cooperative settings to manage moisture and labor constraints	[126]
Solid-state fermentation (SSF)	Dewatered pulp / bagasse	Biotransformation, preservation, digestibility improvement, N enrichment	Can improve apparent protein and alter fiber degradation, but quantitative dairy feeding comparisons remain limited	Heat build-up, uneven moisture, inconsistent inoculum performance, scale-up difficulty	Technically promising but more management-intensive than standard ensiling	[127]
Emerging hybrid technologies (ultrasound, enzyme–microbial combinations, pretreatment-assisted drying)	Selected liquid, slurry, or bagasse streams	Faster detoxification and/or dehydration	Ultrasonic pretreatment reduced hydrogen cyanide by about 40.36% and cyanogenic glycosides by about 24.95% in cassava juice under optimized conditions	Limited feed-scale validation; equipment cost; uncertain field adoption	Promising research direction, but not yet routine for farm/cooperative application	[128]

LAB = Lactic acid bacteria, CP = Crude protein, NPN = Non-protein nitrogen, QC = Quality control, HCN = Hydrogen cyanide, SSF = Solid-state fermentation. Apparent CP refers to crude protein estimated from total nitrogen (N × 6.25) and may include substantial NPN, particularly in urea-treated silages.

### Biological/chemical interventions

**Additives operationalize upcycling by addressing predictable bottlenecks in cassava pulp:** low CP, variable availability of fermentable sugars, and the risk of aerobic deterioration during feed-out. Three intervention classes recur in the recent literature:

**Inoculants (LAB; homo- and heterofermentative):** LAB can accelerate acidification and suppress undesirable microbes; heterofermentative strains (e.g., *Lentilactobacillus* *buchneri*) are frequently used to improve aerobic stability via higher acetic acid production, though effects are substrate- and management-dependent [120].

**Nitrogen sources (urea or other N inputs):** Urea addition can raise measured CP (largely as NPN) and support fermentation dynamics, but requires careful dosing and mixing uniformity; cassava pulp silage trials with urea and molasses show consistent effects on chemical composition and fermentation outputs, reinforcing the need to report N source and inclusion explicitly [24].

**Carbohydrate additions (molasses) and mixed-substrate silages:** Molasses can support more robust lactic fermentation (particularly when substrate water-soluble carbohydrate is limiting) and may reduce DM losses in some systems; mixed silages (e.g., cassava foliage with grasses plus LAB/molasses) demonstrate that co-ensiling can stabilize moisture and fermentation while rebalancing nutrient profiles [126].

From an implementation standpoint, these interventions should be treated as specification tools: cooperatives can standardize recipes based on incoming DM and target aerobic stability, rather than leaving additive use to informal practice.

### Solid-state fermentation (SSF) and other bioconversion routes

Where evidence and infrastructure allow, non-silage fermentation can be used to upcycle cassava residues by protein enrichment (microbial biomass) and reduction of antinutritional factors. SSF has drawn renewed attention because it can operate under low free-water conditions (compatible with many agro-residues) and simultaneously enhance digestibility and increase apparent protein content through microbial growth. A review synthesizes microorganisms, process factors, and product opportunities for food and feed applications, positioning SSF as a platform for transforming cassava residues rather than simply preserving them [127]. Sector-specific synthesis also notes SSF as a suitable approach for detoxification and protein enrichment of cassava waste, while emphasizing constraints such as process control and scale-up [53]. While some protein enrichment studies exist using yeasts (e.g., *Saccharomyces cerevisiae* and related systems), the translational gap for dairy remains the need to (i) quantify true-protein vs. NPN, (ii) characterize fermentability impacts on rumen function, and (iii) evaluate cost and safety controls at cooperative scale, areas that can form a focused research agenda [133].

### Emerging low-cost or hybrid technologies

Emerging low-cost or hybrid technologies also merit brief discussion. Ultrasonic pretreatment has been reported to reduce HCN by about 40.36% and cyanogenic glycosides by about 24.95% in cassava juice within 10 min under optimized laboratory conditions, illustrating proof-of-concept potential for rapid detoxification. Likewise, enzyme–microbial combinations, such as cellulase plus cyanide-utilizing bacteria, have outperformed untreated ensiling in reducing cyanide. Traditional starter-controlled fermentation systems are also conceptually relevant because they reinforce the broader principle that controlled inoculation is preferable to uncontrolled fermentation when cyanide reduction and batch stability are priorities. However, direct dairy feed validation of these emerging and hybrid approaches remains limited [15, 128].

**AEROBIC STABILITY AFTER OPENING:** RELEVANCE FOR SMALLHOLDER DAIRY AND COOPERATIVE SYSTEMS

For cooperatives and smallholders, the last meter of upcycling is often feed-out, where aerobic exposure can rapidly erode the value of an otherwise well-made silage. Aerobic deterioration reduces palatability and increases the risk of yeast/mold proliferation; it is therefore a production and safety issue, not merely a storage nuisance. Recent dairy silage management research highlights feed-out rate and face management as practical levers to reduce aerobic deterioration in bunker systems, reinforcing the need to treat cooperative standard operating procedures (fast filling, adequate packing, effective sealing, and disciplined feed-out) as critical control points [134]. At the additive level, modern silage literature consistently links improved aerobic stability to fermentation profiles (often higher acetic acid with some heterofermentative LAB), but also shows that inoculant effects are not universal, hence the importance of aligning inoculant strategy with substrate type, DM, and management [120]. Cassava pulp silage studies that explicitly measured aerobic stability (including additive factorial designs) provide a template for cooperative standards (e.g., minimum stability hours under standardized exposure tests), and they illustrate that aerobic stability can be engineered, rather than assumed, through additive choice and fermentation management [24].

Aerobic stability after opening should be monitored using practical indicators rather than by visual assessment alone. A widely used operational definition of failure is the time required for silage temperature to rise more than 2°C above ambient temperature, because this increase reflects renewed aerobic microbial activity and the onset of deterioration. In cassava pulp silage, pH values below about 4.0 are generally consistent with satisfactory preservation at silo opening, but pH should be interpreted alongside post-opening stability, as yeasts can metabolize organic acids after air exposure, leading to heating and a secondary rise in pH. This process is especially important in wet cassava pulp, where early heating of the pile should be regarded as a warning sign of yeast activity rather than a benign temperature fluctuation. Heterofermentative microorganisms may improve aerobic stability by increasing antifungal fermentation products, particularly acetic acid and, in some cases, propionic acid, which act as natural preservatives by inhibiting yeasts and molds. Accordingly, practical quality control after opening should include the opening pH, the temperature rise relative to ambient, and the number of hours before instability develops, especially under humid tropical conditions, where repeated air ingress and slow feed-out can accelerate spoilage [24, 116, 135, 136].

**RATION LEVEL INTEGRATION IN LACTATING COWS:** PERFORMANCE, RUMEN FUNCTION, AND MILK COMPONENTS

**Cassava residues as energy sources:** fermentability considerations vs. cereal grains

At the ration level, cassava pulp–derived ingredients should be treated primarily as energy sources that can partially substitute cereal grains, but with fermentability characteristics that may differ from maize depending on (i) the residual starch-to-fiber ratio of the specific pulp stream and (ii) the processing route (fresh/wet, dried, ensiled, fermented). *In vitro* evidence indicates that replacing crushed maize with cassava residue can shift fermentation characteristics, underscoring that the substitution is not “isocaloric by default” and that the response depends on inclusion level and the background diet [137]. For fermented or ensiled cassava pulp products, recent work highlights that additive-driven processing (e.g., LAB + molasses + urea) can alter gas kinetics, digestibility indices, and fermentation profiles, reinforcing the need to consider cassava pulp as a family of energy ingredients whose functional value depends on process specification rather than ingredient name [35]. Practically, this means that substituting cereals with cassava residues should be approached through fermentability management (rate and extent of rumen carbohydrate degradation) rather than crude starch equivalence, particularly in high-concentrate lactation diets.

### Rumen health constraints

The principal biological constraint when integrating cassava residues into lactating cow diets is the risk of SARA if rapidly fermentable carbohydrate supply increases without adequate physically effective fiber and appropriate adaptation. A recent critical review of SARA emphasizes that the condition arises from prolonged ruminal pH depression, driven by excessive intake of rapidly fermentable carbohydrates combined with insufficient effective fiber, and details management implications at the diet and feeding system levels [138]. Empirically, a study demonstrates that increasing physically effective NDF in high-concentrate diets can improve chewing activity and rumen conditions in ways consistent with reduced SARA risk, supporting physically effective NDF as a practical control lever when replacing cereal grains with alternative fermentable carbohydrates [93]. Therefore, cassava pulp inclusion should be paired with explicit controls on (i) forage and physically effective NDF supply, (ii) feed delivery consistency (avoid slug feeding of high-fermentability components), and (iii) step-up adaptation during ration transitions, because the margin for error is smallest in early and peak lactation. When cassava pulp is fed in ensiled form, feed-out practices that preserve aerobic stability also indirectly support rumen health by maintaining palatability and stabilizing feed intake [24].

From a field perspective, adaptation and monitoring should be stated explicitly. When cassava residues replace cereal energy sources, inclusion should be increased gradually and interpreted together with rumination behavior, manure characteristics, fecal starch, and milk component responses. The milk fat-to-protein ratio can be a useful non-invasive herd-level indicator, but recent review evidence cautions against using it alone to diagnose ruminal acidosis. In a recent critical review, a milk fat-to-protein ratio threshold of about 0.81 showed specificity for SARA, whereas broader field-oriented guidance continues to use low milk fat-to-protein ratio values, approximately 1.0–1.1 or lower, as warning signals for possible MFD or SARA when combined with other indicators [139, 140].

Additional practical indicators should therefore be mentioned. Reduced rumination time, loose or bubbly manure, and elevated fecal starch are consistent with excessive fermentability and impaired total-tract starch utilization. Fecal starch is especially useful because it is quantitatively related to total-tract starch digestibility: in lactating dairy cows, each 1 percentage-unit increase in fecal starch corresponds to about a 1.2–1.25 percentage-unit decrease in total-tract starch digestibility. Thus, when cassava residues are used to replace cereal energy, herd-level interpretation is strongest when milk fat-to-protein ratio, rumination behavior, manure consistency, and fecal starch are evaluated together rather than in isolation [141, 142].

### Protein–energy synchrony and implications for microbial protein synthesis

Cassava pulp streams are typically low in CP, so their use as energy substitutes can inadvertently reduce rumen-degradable nitrogen supply unless the ration is rebalanced. This matters because maximal microbial protein synthesis depends on the temporal alignment of rumen-available energy and nitrogen, especially in diets containing substantial rapidly fermentable carbohydrates. Recent *in vivo* work in lactating Holstein cows tested dietary strategies centered on the balance of rumen-degradable starch and rumen-degradable protein and evaluated their impacts on digestibility, rumen fermentation, nitrogen partitioning, and lactation performance, highlighting both the potential value and the practical complexity of synchrony-based formulation [143]. In cassava systems, fermentation/bioconversion routes have been explored as an enabling technology to address the low CP constraint: a lactating cow trial using fermented cassava pulp with yeast waste under different roughage-to-concentrate ratios reported effects on rumen fermentation, nutrient digestibility, and milk production outcomes, illustrating that upcycled cassava pulp may function differently from untreated pulp at the same nominal inclusion [23]. At the broader cattle level, a systematic review and meta-analysis evaluating yeast-fermented cassava as a protein source further supports the concept that microbial enrichment can improve performance-related endpoints, while also signaling the need to distinguish increases in measured CP due to true microbial protein versus shifts toward NPN depending on the fermentation strategy used [144]. Recent evidence from dairy helps clarify this point. In mid-lactation cows fed fermented cassava pulp with yeast waste, ruminal ammonia-N increased and milk protein rose from 3.05% to 3.25% without depressing milk yield, supporting improved rumen nitrogen availability but not directly proving improved post-ruminal amino acid supply. Likewise, in tropical lactating cows fed fermented TMR based on fresh cassava root with sulfur and urea, higher sulfur increased bacterial population by 6.1%, propionate by 4.6%, and markers of microbial CP synthesis, including allantoin absorption and microbial CP. A broader meta-analysis of yeast-fermented cassava in cattle further reported higher ruminal levels of volatile fatty acids and propionate, and, in lactating cows, increased milk yield by 1.02 kg/day, along with increases of 7.4% in milk fat, 6.3% in milk protein, and 2.8% in lactose. Nevertheless, these responses should still be interpreted primarily as evidence of improved rumen nitrogen capture and microbial protein synthesis efficiency rather than as direct proof of improved amino acid profile, because true-protein and amino acid-flow data remain scarce [23, 144, 145].

### Mineral and byproduct-associated issues

Mineral-related constraints are most often driven not by intrinsic mineral richness but by extraneous ash/soil contamination, which dilutes energy density and can introduce mineral antagonism issues. Authoritative forage-quality guidance from the U.S. Department of Agriculture, Agricultural Research Service notes that elevated ash can indicate soil contamination and that each percentage point of soil contamination can translate into a meaningful loss of energy value, an insight directly relevant to cassava residues that are dried or handled under contamination-prone conditions [146]. Because cassava pulp is frequently produced and stored in wet form, contamination risk may also arise from contact with soil during handling or from poor drying practices; cooperative-level specifications should therefore include total ash and insoluble ash as routine indicators, alongside physical screening for foreign matter [147]. In addition, cassava residue supply chains in humid environments warrant integration into broader contaminant surveillance (e.g., mycotoxins), but at the ration level the most actionable control is to ensure the ingredient’s hygienic quality (no visible spoilage, stable silage at feed-out) and to avoid using suspect batches in lactating groups with high intake and high sensitivity to feed quality fluctuations [24]. Milk safety and quality should also be interpreted more explicitly. Recent reviews indicate that carryover of aflatoxin B1 from feed into AFM1 in milk is typically 1–2%, but may reach 6–6.5% in high-yielding cows, meaning that contaminated feed can result in non-compliant milk even in the absence of obvious clinical signs. In cassava-based feeding systems, cyanogenic exposure is also relevant because HCN is detoxified to thiocyanate, which is partly excreted in milk. In tropical lactating cows fed fresh cassava root in fermented TMR with sulfur and urea, blood thiocyanate increased by 21.6% and milk thiocyanate concentration also increased, while somatic cell count decreased by 18.3% at the higher sulfur level. Similarly, supplementation with fresh cassava peel increased milk thiocyanate and improved hygienic quality without depressing milk yield or milk composition [110, 145].

### Expected production responses

When cassava residues are used as energy sources in balanced diets, available evidence suggests that lactation performance can be maintained, but responses are contingent on ration structure and rumen stability. In lactating cows, feeding trials using fermented cassava pulp systems have documented measurable changes in rumen fermentation and digestibility with corresponding milk production outcomes, providing direct evidence that cassava pulp can be integrated into lactation diets without necessarily compromising milk yield when formulation and roughage-to-concentrate ratio are managed [23]. From a milk component perspective, the dominant risk is not a universal decline in milk yield but rather MFD under conditions that promote low rumen pH and altered biohydrogenation pathways. Recent reviews synthesize that diet-induced MFD is linked to disruptions in rumen fermentation and shifts in fatty acid biohydrogenation, and they emphasize prevention through ration formulation and fiber management, exactly the control levers implicated when replacing cereals with highly fermentable by-products [148]. Thus, in practical monitoring, cooperatives should treat milk fat percentage (and trends in fat-to-protein ratio), DM intake stability, rumination activity, fecal consistency, and body condition dynamics (body weight/body condition score) as early warning indicators of whether cassava residue inclusion is functioning as intended. Evidence on physically effective NDF-based mitigation further supports using fiber effectiveness as a central design and monitoring variable, rather than relying solely on NDF concentration targets [93].

**CIRCULARITY AND SYSTEMS IMPLICATIONS CIRCULAR FEED RATIONALE:** REDUCING WASTE, LOWERING FEED–FOOD COMPETITION

Integrating cassava processing residues into dairy rations fits squarely within a circular feed logic: ruminants can convert human-inedible biomass streams into milk, thereby reducing disposal burdens and partially substituting conventional concentrates that compete more directly with human food supply. Recent circularity syntheses in the feed chain explicitly position industrial co-products and former foodstuffs as priority circular resources (as opposed to “recovered feed from waste,” which is often legally constrained), underscoring that circularity gains are strongest when the feed material is a byproduct already generated by food/industrial processing rather than a dedicated feed crop expansion [149]. In cassava value chains, this framing is particularly relevant because large volumes of solid residues and wastewater are routinely produced, and the choice of valorization pathway determines whether the system yields a food-grade starch product plus a circular feed stream or shifts residues toward energy/industrial uses [29]. However, cassava circularity should be evaluated as a decision among competing valorization routes rather than as an automatically positive attribute of residue use. Recent cassava-specific life cycle and techno-economic evidence indicates that the preferred pathway depends strongly on residue type, moisture content, transport distance, stabilization energy, and the form of energy recovery. In a 2025 study of cassava-based ethanol production, three stillage-management scenarios were compared: drying wet distillers’ grains with solubles for sale as feed; anaerobic digestion of stillage for biogas used for heat; and biogas use in combined heat and power. The combined heat and power scenario performed best in terms of environmental and economic performance, achieving a net global warming potential of −1515.05 kg CO₂-eq per ton of ethanol and the highest profit of USD 396.80 per ton of ethanol. These results indicate that, at least for very wet industrial stillage, energy recovery may outperform feed-drying pathways when internal energy substitution and surplus electricity generation are possible [150].

## PRACTICAL SYSTEM BOUNDARIES

Biorefinery/life cycle assessment reviews emphasize that cassava residues can be directed to multiple products (animal feed, biogas/bioenergy, volatile fatty acids/biochemicals, fertilizers), and that environmental outcomes are highly sensitive to system definition, substitution assumptions, and coproduct allocation [29]. A useful decision boundary is therefore: feed first when (i) residue quality can be stabilized and controlled, (ii) local dairy demand exists within a viable transport radius, and (iii) the alternative use is low-value disposal or low-efficiency energy recovery; whereas energy/industrial routes may dominate when residues are too dilute or contaminated for safe feeding, when dairy demand is distant, or when wastewater streams (often the largest residue by volume) are better suited to anaerobic digestion or volatile fatty acid platforms than to feed [151]. Importantly, emerging analyses warn against assuming universal climate benefits for waste-to-feed without robust, context-specific accounting, reinforcing the need to avoid overgeneralization and to report decision boundaries transparently [152]. Recent cassava starch and cassava-biorefinery studies make these system boundaries more concrete. In a 2026 factory-level study of a 120 t/day cassava starch plant in Vietnam, shifting excess biogas from a base case used mainly for drying to a biogas-to-electricity configuration reduced total fossil energy use from 3730.7 to 2901.7 MJ per ton and greenhouse gas emissions from 0.79 to 0.72 tCO₂-eq per ton. The same study reported annual production-cost savings of about USD 0.12 million, a payback period of 6.6 years, and a return on investment of 15%, illustrating that circularity claims are highly sensitive to energy-integration choices rather than to residue valorization alone [153].

## COST DRIVERS

Economically, cassava pulp upcycling for dairy is dominated by logistics and stabilization costs, not by raw material purchase price alone. First, transport cost per unit of DM is the principal bottleneck for wet pulp streams; as moisture increases, the system pays to move water, making distance-to-plant and backhaul logistics decisive. Second, the drying-versus-ensiling choice is fundamentally a trade-off between energy/capital costs (drying infrastructure, fuel, labor) and spoilage/handling risk (ensiling demands compaction, sealing, feed-out discipline, and additive budgets). Third, storage losses (DM shrink, aerobic spoilage) can erase apparent feed-cost savings; recent silage microbiology work emphasizes that aerobic deterioration during feed-out is a predictable failure mode unless management and inoculant strategy are aligned with substrate properties [53]. Finally, circular feeds require quality control costs that are often omitted from economic comparisons: basic DM/ash screening, periodic mycotoxin surveillance (risk-based), optional cyanide testing triggers, and recordkeeping are part of the true cost of “making residues feed-grade,” and Codex guidance for cassava matrixes explicitly foregrounds prevention and control measures across handling and storage as necessary risk management steps (even when the end use is feed) [112]. Available cassava-specific case evidence also suggests that circularity pathways are sensitive to local infrastructure and awareness. Among 240 cassava processors in Nigeria, current waste management was dominated by dumping, runoff, and burning, while awareness of biogas technology was only 21.25%; however, willingness to adopt biogas technology rose to 74.17% after awareness creation. These results suggest that economic feasibility is shaped not only by engineering costs but also by local management capacity, access to technology, and whether residues can be moved into higher value uses at all [154].

## KEY DATA GAPS FOR LIFE CYCLE ASSESSMENT AND POLICY-RELEVANT METRICS

The life cycle assessment evidence base for cassava residue valorization remains fragmented, with reviews highlighting recurring methodological challenges: inconsistent functional units (per ton residue vs. per kg milk), weak primary data on residue handling emissions and losses, uncertain allocation across starch (main product) and residue streams, and highly assumption-sensitive substitution credits (what feed or energy is “displaced”) [29]. For dairy-focused claims, the most critical gaps are (i) farm-level measurements linking cassava residue inclusion to changes in enteric methane intensity, manure emissions, and milk composition under realistic rations, (ii) storage and processing inventories (electricity/fuel for drying, additive production footprints, shrink losses), and (iii) risk management externalities (cost and environmental impacts of rejected batches, detoxification steps, and quality testing) [152]. Policy-relevant interpretation also requires clarity on legal feasibility and governance: circular feed briefings note that some circular resources are permitted while others are constrained, and that regulatory alignment between food and feed systems shapes what circular pathways can scale in practice [149]. This policy point can be stated more concretely. In the European Union, materials placed on the market as feed are governed by feed-marketing legislation and the Catalog of Feed Materials. When a feed material is not yet listed in the Catalog, its first use on the market must be notified through the Feed Materials Register. This illustrates that residue streams can function legally as feed materials when they are specified, placed on the market, and controlled as such, rather than remaining undefined “waste.” By contrast, we did not identify clear jurisdiction-wide legal maximum inclusion limits for cassava pulp in dairy cows; current practical guidance is still driven more by feeding trials than by formal feed-law inclusion caps. Accordingly, the most defensible position for this review is that cassava residues can support circularity and potentially reduce feed–food competition when converted into specification-controlled feed ingredients, but robust claims about net climate benefits or system-wide sustainability should be framed as conditional on context-specific life cycle assessment with transparent boundaries and high-quality primary data.

## CONCLUSION

Cassava processing residues present a strategically important circular feed opportunity for tropical dairy systems, but their successful use depends less on ingredient substitution and more on stream-specific governance. The evidence reviewed here supports four overarching conclusions.

First, cassava waste must be treated as a set of defined residue streams, not a single feed ingredient. Differences in origin (post–starch extraction pulp versus peels or mixed residues), physical form (wet, dried, pelleted, ensiled, fermented), and handling conditions create substantial variation in fermentability, nutrient density, and hazard exposure. Without explicit stream identification and standardized analytical reporting (DM basis, starch and fiber methods, particle size and peNDF descriptors, and, when relevant, cyanide metrics), cross-study comparison and safe formulation guidance remain intrinsically limited. Accordingly, future work should prioritize standardized residue characterization and minimum reporting standards so that results can be compared across studies, production systems, and stabilization pathways with greater confidence.

Second, the two dominant constraints to adoption are stability and safety, and both are controllable when converted into process specifications. High moisture cassava pulp is inherently perishable, making rapid stabilization essential; ensiling is often the most feasible pathway for cooperatives, provided compaction, sealing, and feed-out discipline are standardized and verified. In parallel, cyanogenic risk is best framed as a process-controlled hazard rather than a fixed ingredient property. Where cyanogenic potential is relevant (e.g., peel contamination or high-risk cassava sources), detoxification and monitoring must be integrated into the preservation route, with risk-based testing triggers and clear batch disposition rules. The next research step is therefore not only to test whether a preservation route works under experimental conditions, but also to determine whether it remains reliable under multi-batch, multi-site, and humid tropical field conditions with full hazard surveillance and realistic feed-out pressures.

Third, ration level integration for lactating cows should be designed around fermentability management, rumen health protection, and protein–energy synchrony. Cassava pulp and related ingredients can function as effective energy sources under balanced rations, but their variable starch–fiber architecture means that substitution decisions must be anchored to peNDF adequacy, gradual adaptation, and explicit nitrogen supply adjustments to maintain microbial protein synthesis. In practice, the most sensitive early warning endpoints for implementation are intake stability, rumination activity, fecal indicators of starch escape, and milk component shifts, particularly MFD patterns, rather than milk yield alone. This also implies a clear research priority: future multi-site and multi-year trials in high-producing herds should combine continuous rumen pH monitoring with practical field indicators such as milk fat-to-protein ratio, fecal starch, manure consistency, and hazard surveillance at feed and milk level.

Fourth, the dairy cow evidence base is promising but not yet sufficient for broad generalization to high-producing, high-concentrate temperate systems. Existing trials are geographically concentrated and often short in duration, with limited continuous rumen pH measurement and inconsistent reporting of stream identity, processing, and hazard surveillance. As a result, confidence is highest for cassava-derived energy feeds in the specific contexts studied, while uncertainty remains substantial for true cassava pulp (starch residue) used as a primary energy ingredient in high-yield lactation diets and for long-term health, reproduction, and milk safety outcomes under commercial conditions. To strengthen the evidence base, future studies should move beyond short-term performance outcomes and incorporate integrated assessments linking stabilization method, safety, economics, animal welfare, enteric methane, manure-related emissions, and nutrient cycling.

**Collectively, these findings justify a clear implementation message:** cassava waste can be scaled as a circular ingredient for dairy only when it is managed as a specification-controlled product, with defined streams, validated stabilization routes, and cooperative-level QC/mini-HACCP governance, rather than as an opportunistic byproduct. The next research frontier is therefore not simply more feeding trials, but integrated, multi-site cooperative studies that couple standardized residue definitions, process-controlled stabilization (ensiling and/or bioconversion), and pragmatic monitoring systems to produce adoption-ready evidence on performance, safety, and economics. In parallel, translational progress will depend on practical decision-support tools for cooperatives and smallholders, including simple procurement specifications, low-cost stabilization options, and batch-level quality control indicators that can be applied under routine field conditions.

Strengths of the current evidence base include consistent demonstration of improved preservation and partial detoxification through ensiling and microbial interventions, as well as successful integration into smallholder and cooperative feeding systems.

Limitations include high compositional variability across streams and processing methods, limited long-term data from high-producing commercial herds, and insufficient integration of life cycle assessment with on-farm performance and milk safety outcomes.

Future scope should prioritize multi-site, long-term feeding trials that combine standardized residue characterization, process-controlled stabilization, and integrated economic/life cycle assessments. Development of practical cooperative guidelines and policy frameworks supporting specification-controlled circular feeds will be essential for wider adoption.

In conclusion, cassava processing residues can meaningfully contribute to sustainable dairy production by reducing feed costs, minimizing waste, and enhancing circularity. Their successful incorporation requires moving beyond opportunistic use toward specification-controlled, process-managed feed ingredients. With continued research and practical implementation frameworks, these abundant by-products can support more resilient and environmentally responsible tropical dairy systems.

## DATA AVAILABILITY

The data generated during the study are included in the manuscript. 

## GENERATIVE AI DECLARATION

The authors declare that generative artificial intelligence (AI) tools were used solely to improve language, grammar, and readability during manuscript preparation. All scientific content, data analysis, interpretation of results, and conclusions were developed and verified by the authors. The authors take full responsibility for the accuracy, integrity, and originality of the work presented, and no AI tool was listed as an author.

## AUTHORS’ CONTRIBUTIONS

BW: Conceptualized the review, designed the review framework, conducted the literature search, interpreted the evidence, drafted the manuscript, and approved the final version. SS: Conceptualized the review, designed the methodology, curated the literature, interpreted the evidence, drafted the manuscript, and supervised the study. AN: Contributed to the review methodology, interpreted the evidence, critically revised the manuscript, and approved the final version. TT: Critically reviewed and edited the manuscript. AYS and JN: Contributed to literature interpretation and critically reviewed and edited the manuscript. All authors have read and approved the final manuscript.
